# *Arabidopsis thaliana—Myzus persicae* interaction: shaping the understanding of plant defense against phloem-feeding aphids

**DOI:** 10.3389/fpls.2013.00213

**Published:** 2013-07-01

**Authors:** Joe Louis, Jyoti Shah

**Affiliations:** ^1^Department of Entomology and Center for Chemical Ecology, The Pennsylvania State UniversityUniversity Park, PA, USA; ^2^Department of Biological Sciences, University of North TexasDenton, TX, USA

**Keywords:** green peach aphid, effectors, Hemiptera, phloem-feeding insect, plant defense mechanisms, susceptibility factors

## Abstract

The phloem provides a unique niche for several organisms. Aphids are a large group of Hemipteran insects that utilize stylets present in their mouthparts to pierce sieve elements and drink large volumes of phloem sap. In addition, many aphids also vector viral diseases. *Myzus persicae*, commonly known as the green peach aphid (GPA), is an important pest of a large variety of plants that includes *Arabidopsis thaliana*. This review summarizes recent studies that have exploited the compatible interaction between Arabidopsis and GPA to understand the molecular and physiological mechanisms utilized by plants to control aphid infestation, as well as genes and mechanisms that contribute to susceptibility. In addition, recent efforts to identify aphid-delivered elicitors of plant defenses and novel aphid salivary components that facilitate infestation are also discussed.

## Introduction

The phloem, which provides a conduit for resource distribution and signaling, also provides a niche for some organisms. However, for these organisms the phloem also provides several challenges in that the phloem sap is under high pressure, has a high C:N ratio and a high osmolarity due to elevated sugar content. Furthermore, when ruptured or punctured the sieve elements are prone to occlusion. Aphids (Hemiptera: Aphididae) constitute a large group of “piercing-sucking” class of insects that have adapted to feeding from sieve elements (Pollard, 1973; Blackman and Eastop, 2000; Walling, 2000). Nearly 250 amongst the ~4000 aphid species that have been described are considered as pests (Dixon, 1998; Blackman and Eastop, 2000). Damage to the plant results from loss of phloem sap and changes in source-sink patterns as a consequence of which nutrient flow to the primary growth zones is reduced (Mittler and Sylvester, 1961; Girousse et al., 2005). Some aphids also vector viral diseases of plants, thereby causing further loss of plant productivity and quality (Kennedy et al., 1962; Matthews, 1991; Dixon, 1998). Viral infection in the host plant can further influence severity of aphid infestation (Ziebell et al., 2011; Gutiérrez et al., 2013).

**Figure 1:**
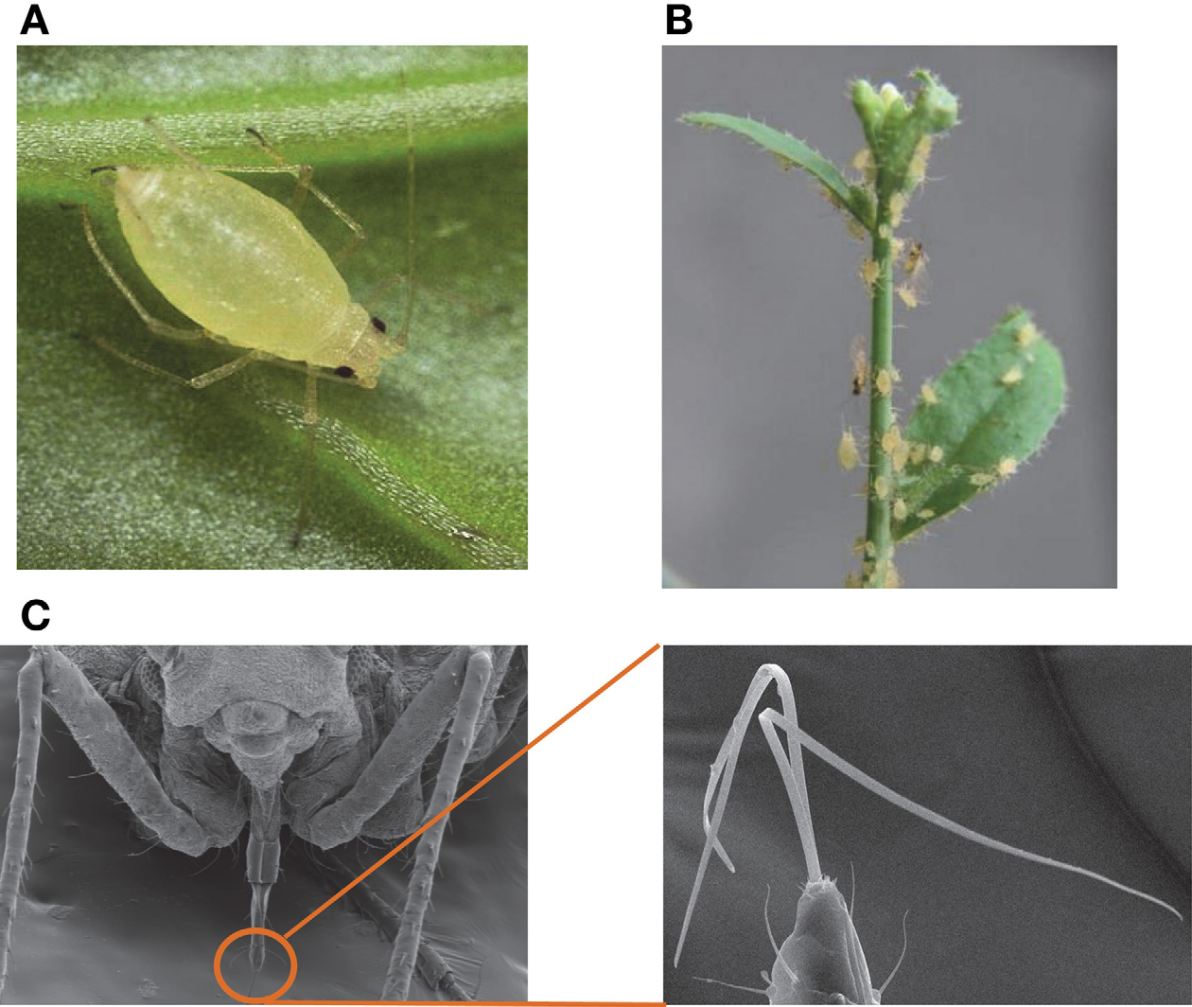
**Green peach aphid on Arabidopsis. (A)** Green peach aphid on Chinese cabbage (*Brassica rapa* var *chinensis*). Images by Nick Sloff. **(B)** Green peach aphid on *Arabidopsis thaliana*. **(C)** Mouthparts of aphid. Left panel: SEM image of showing aphid mouthpart; Right panel: Aphid stylet. Images provided by John Diaz-Montano. The above images were adapted with permission from Louis et al. (2012c) *Arabidopsis thaliana*—Aphid Interaction. The Arabidopsis Book (First published on May 22, 2012:e0159. doi: 10.1199/tab.0159). Copyright American Society of Plant Biologists (thearabidopsisbook.org).

Aphids can be broadly classified as specialists or generalists (Lankau, 2007). Specialists like the cabbage aphid (*Brevicoryne brassicae*) and the mustard aphid (*Lipaphis erysimi*) have a limited host range that is restricted to cruciferous plants. In contrast, as described below, a generalist like the green peach aphid (GPA; *Myzus persicae* Sulzer) (Figures 1A,B) feeds on a large variety of plants belonging to different families (Blackman and Eastop, 2000; Lankau, 2007). The mouthparts of aphids are modified into slender stylets (Figure 1C), which enable penetration of the sieve element to consume phloem sap. Present within each stylet is a salivary canal through which saliva is released into the plant tissue, and a food canal through which the insect uptakes phloem sap. The predominantly intercellular route taken by stylets, combined with the activity of aphid salivary components, minimizes physical damage to the plant tissue, thus averting substantial wounding-related responses from the plant (Miles, 1999; Walling, 2000; Tjallingii, 2006). In addition, the aphid saliva also contains factors that have been suggested to prevent or reverse sieve element occlusion (SEO) (Will et al., 2007, 2009). The aphid stylets occasionally may pierce host cells, seemingly to ingest/sample minute amounts of plant material (Tjallingii, 1990, 2006). These interactions of the aphid stylets with the sieve elements and plant cells also provide an interface for exchange of metabolites and macromolecules between the aphid and the plant that potentially could promote or deter colonization.

### Green peach aphid

The host range of the GPA comprises over 400 plant species belonging to nearly 50 plant families, including important crops like potato and sugar beet, stone fruits (e.g., peach, almond, and cherry), and horticultural crops in the Brassicaceae, Solanaceae and Cucurbitaceae families (Blackman and Eastop, 2000). More than 100 viral diseases are vectored by GPA, which is the most important virus vector on vegetable crops (Kennedy et al., 1962; Matthews, 1991). These characteristics, combined with the capacity of the GPA population to rapidly increase in size and GPA's resistance to a large number of insecticides (Georghiou and Lagunes-Tejada, 1991; Vasquez, 1995; Devonshire et al., 1998; Silva et al., 2012a,b) has resulted in GPA being categorized amongst the top three agricultural pests in the USA (Klein and Waterhouse, 2000).

GPA is capable of sexual and asexual (parthenocarpic) reproduction. Asexual reproduction is characterized by a telescopic generation in which an adult female contains embryos that themselves contain embryos. In nature, the GPAs life cycle includes primary and secondary hosts. The sexual cycle is completed on the primary host, which comprise stone fruits like peach (*Prunus persicae*), Canadian plum (*P. nigra*), black cherry (*P. serotina*), and dwarf Russian almond (*P. tenella*) (Blackman and Eastop, 2000). Secondary hosts include a large variety of plants, including potato, tomato, eggplant, lettuce, celery, mustard, cabbage, radish, and squash (Blackman and Eastop, 2000).

The near completion of the GPA genome sequence (http://tools.genouest.org/tools/myzus/; Ramsey et al., 2007) combined with the development of plant-delivered RNA-interference (RNAi) technology for gene silencing in GPA (Pitino et al., 2011; Pitino and Hogenhout, 2013), have made available new genomic resources and powerful reverse-genetic tools that have begun to facilitate understanding the function of GPA genes and their contribution to plant-aphid interaction.

#### Salivary effectors promote insect performance on plant

Once the aphid initiates feeding on the host plant it delivers salivary secretions into the plant tissue, which potentially allow the aphid to circumvent plant defenses. Aphids produce two kinds of salivary secretions: gelling saliva and watery saliva (Miles, 1999). The gelling saliva, which is secreted when the stylet is penetrating the host tissue but outside the sieve element, forms a sheath around the stylet that likely facilitates stylet movement through the plant tissue and minimizes damage to the stylet tip. In addition, the salivary sheath helps rapidly seal the wound caused by aphid stylet penetration, thus minimizing the likelihood of wounding-response activation by the host. On the other hand, the watery saliva, which is intermittently released in the plant tissue when the insect is feeding, contains factors that enable the aphid to prevent and maybe also reverse phloem occlusion, thus allowing the insect to feed continuously from a single sieve element (Miles, 1999; Will et al., 2007, 2009). The saliva also contain effectors that manipulate host physiology to facilitate colonization (Rodriguez and Bos, 2013). Publicly available salivary gland ESTs have been utilized to identify GPA effectors that promote colonization (Bos et al., 2010). The salivary protein MpC002 when transiently expressed in *Nicotiana benthamiana* or expressed in transgenic *Arabidopsis thaliana* was found to enhance GPA colonization (Bos et al., 2010; Pitino and Hogenhout, 2013). By contrast, when MpC002 expression in the GPA was silenced by allowing insects to feed on *N. benthamiana* leaves that were transiently expressing a dsRNA construct, or in transgenic Arabidopsis stably expressing dsRNA, insect fecundity was significantly reduced (Pitino et al., 2011; Pitino and Hogenhout, 2013), thus indicating that the MpC002 facilitates infestation. Similarly, in pea aphid (*Acyrthosiphon pisum*) silencing of the homologous *C002* gene had detrimental effects on the insect's ability to colonize plants (Mutti et al., 2008). However, unlike *MpC002*, expression of the pea aphid *C002* in Arabidopsis had no effect on GPA fecundity, suggesting specificity in the role of these orthologous genes in promoting aphid infestation (Pitino and Hogenhout, 2013).

*PIntO1* (*Progeny Increase to Overexpression 1*; also known as *Mp1*) and *PIntO2* are two other putative salivary protein-encoding GPA genes that have been suggested to facilitate GPA colonization on Arabidopsis. Compared to non-transgenic plants, the number of nymphs produced was larger on transgenic plants expressing *PIntO1* or *PIntO2* from the phloem-specific *AtSUC2* promoter (Pitino and Hogenhout, 2013). However, GPA colonization was not impacted when the pea aphid homologs (*ApPIntO1* and *ApPIntO2*) were similarly expressed in Arabidopsis (Pitino and Hogenhout, 2013), thus suggesting that the GPA PIntO1 and PIntO2 proteins specifically target an Arabidopsis factor/mechanism that is involved in promoting GPA fecundity. Plant-delivered RNAi silencing of *PIntO2* expression in GPA had a detrimental effect on the insect's ability to replicate on Arabidopsis, further indicating that PIntO2, which is delivered into the plant by the GPA, is essential for promoting insect multiplication on Arabidopsis. Although the biochemical function of MpC002, PIntO1, and PIntO2 are not known, the above studies indicate that the GPA saliva contains factors that likely manipulate host physiology, thus allowing the insect to better adapt to the host plant and promote reproduction.

## The *arabidopsis thaliana*-green peach aphid pathosystem: a model system for understanding plant defense and susceptibility against aphids

**Figure 2:**
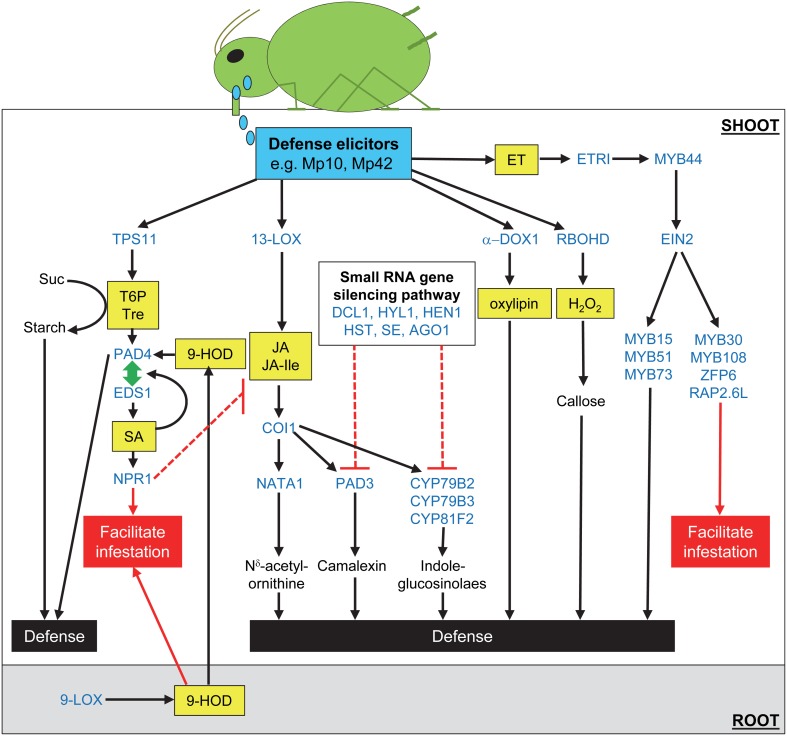
**Model depicting relationship between genes and mechanisms that influence Arabidopsis interaction with the green peach aphid.** Green peach aphid (GPA) salivary secretions contain effectors that promote infestation, as well as elicitors (e.g., Mp10 and Mp42) that are recognized by the host to turn on defense responses. GPA infestation on the shoot results in the induction of *LOX5* expression in roots and a concomitant increase in the levels of LOX5-derived oxylipins (e.g., 9-HOD). *LOX5* expression is likely induced by a GPA infestation-induced factor that is translocated from the leaves to the roots. The *LOX5*-derived oxylipins are transported from the roots to the shoots where one or more of these oxylipins stimulate expression of the defense regulatory gene, *PAD4*. A *PAD4*-dependent mechanism adversely impacts GPA settling, feeding and fecundity on Arabidopsis. *PAD4* expression is further stimulated by the trehalose (Tre) metabolic pathway. GPA infestation results in the elevated expression of *TPS11*, which encodes an enzyme with Trehalose-6-phosphate (T6P) synthase and T6P phosphatase activities that is required for promoting *PAD4* expression in GPA-infested plants. *TPS11* also promotes accumulation of starch at the expense of sucrose (Suc), which is a major feeding stimulant, thereby generating a secondary sink that is detrimental to the insect's ability to colonize Arabidopsis. *TPS11* and *PAD4* are also required for accumulation of an antibiosis factor in the petiole exudates that limits insect fecundity. However, the GPA has evolved mechanisms that over time spent on the plant suppress this *TPS11*/*PAD4*-determined antibiosis activity. The GPA has also evolved to utilize one or more of the 9-LOX-derived oxylipins, or products thereof, as cues to stimulate feeding from phloem and xylem, and enhance fecundity. These oxylipins, which are consumed by the insect from the plant, likely induce changes in the GPA gene expression/physiology, thus allowing the insect to overcome and/or bypass plant defenses and adapt to the host plant. Salicylic acid (SA) signaling through *NPR1* is also stimulated in GPA-infested plants. In plant-pathogen interaction, the PAD4 protein functions along with its interacting partner EDS1 in an amplification loop that promotes SA synthesis, leading to activation of SA dependent defenses. SA in turn amplifies *PAD4* and *EDS1* expression, thus resulting in positive amplification of this PAD4/EDS1-SA loop in plant defense against pathogens. Although *EDS1* expression and SA signaling are activated in GPA-infested Arabidopsis, genetic studies confirm that SA and *EDS1* are not required for controlling GPA infestation on Arabidopsis. Quite to the contrary, SA by antagonizing the jasmonic acid (JA; active form is JA-Isoleucine [JA-Ile]) signaling mechanism likely facilitates GPA infestation. JA, which is synthesized by the 13-LOX pathway, is required for controlling severity of GPA infestation. JA promotes the accumulation of N^δ^-acetylornithine, camalexin and indole-glucosinolates, which are detrimental to GPA. Expression of *PAD3*, which is involved in camalexin synthesis and some genes involved in glucosinolate synthesis (e.g., *CYP79B2* and *CYP81F2*) are negatively regulated by the small RNA gene-silencing mechanism involving DCL1, HYL1, HENT1, HST, SE, and AGO1. Oxylipins synthesized by the α DOX1 pathway and reactive oxygen species (ROS) produced by the NADPH oxidase RBOHD are also involved in controlling GPA infestation. H_2_O_2_ promotes callose deposition and thus likely contributes to phloem occlusion and plant defense against GPA. ROS's could also impinge on other signaling/defense mechanisms. Ethylene signaling through ETR1 and EIN2 has also been implicated in Arabidopsis defense against GPA. The ethylene inducible *MYB44* gene is required for controlling GPA infestation. *MYB44* is required for promoting *EIN2* expression in response to harpin treatment, which also induces resistance against GPA in Arabidopsis. The ethylene- and harpin-inducible *MYB15*, *MYB51*, and *MYB73* genes were required for harpin-induced resistance against GPA. By contrast, since mutations in the ethylene- and harpin-inducible *MYB30*, *MYB108*, *ZFP6*, and *RAP2.6L* genes enhanced the effect of harpin on controlling GPA infestation, these genes are shown as factors that facilitate GPA infestation. The relationship between many of these different pathways/mechanisms remains to be studied. All genes/proteins are in blue and signaling molecules are in yellow boxes. Red lines/arrows indicate steps/mechanisms that facilitate GPA infestation, while black lines indicate steps that contribute to defense. Lines ending with a perpendicular bar are indicative of a repressive effect.

Arabidopsis has been used as a model plant by researchers to study plant growth, development and response to stress (Koornneef and Meinke, 2010). Advantages offered by Arabidopsis for molecular-genetic studies include its small size, short generation time, completely sequenced genome and the ease with which it can be transformed (Meinke et al., 1998; Koornneef and Meinke, 2010). The compatible interaction between Arabidopsis and the GPA (Figure 1B) has been successfully utilized to characterize plant response against phloem-feeding insects and to identify plant genes and mechanisms that contribute to defense and susceptibility to these phloem sap-consuming insects (Table 1 and Figure 2) (Louis et al., 2012c). In addition, this pathosystem has also been utilized to study natural genetic variation amongst Arabidopsis accessions to identify quantitative trait loci that influence this interaction (Cabrera y Poch et al., 1998).

**Table 1:** **Arabidopsis mutants that impact green peach aphid colonization**.

**AtG No.**	**Mutant**	**Name/function**	**References**
**DEFENSE SIGNALING**			
At5g05170	*cev1*	*constitutive expression of VSP1*^a^	Ellis et al., 2002a
At2g39940	*coi1*	*coronatine-insensitive1*	Ellis et al., 2002a
At5g03280	*ein2*	*ethylene-insensitive 2*	Kettles et al., 2013
At1g66340	*etr1*	*ethylene response 1*	Mewis et al., 2005, 2006; Kettles et al., 2013
At3g23250	*mby15*	Myb domain protein	Liu et al., 2010
At3g28910	*myb30*	Myb domain protein	Liu et al., 2010
At5g67300	*myb44*	Myb domain protein	Liu et al., 2010
At1g18570	*mby51*	Myb domain protein	Liu et al., 2010
At4g37260	*myb73*	Myb domain protein	Liu et al., 2010
At3g06490	*myb108*	Myb domain protein	Liu et al., 2010
At1g64280	*npr1*	*non-expresser of PR genes1*	Mewis et al., 2005
At3g52430	*pad4*	*phytoalexin-deficient4*^b^	Pegadaraju et al., 2005, 2007; Louis et al., 2012a
At5g13330	*rap2.6L*	AP2 domain protein	Liu et al., 2010
At1g67030	*zfp6*	Zinc-finger protein	Liu et al., 2010
**GLUCOSINOLATE METABOLISM**			
At5g60890	*atr1D*	*altered tryptophan regulation1*	Kim et al., 2008
At4g39950 At2g22330	*cyp79B2 cyp79B3*	Double mutant is deficient in indole-glucosinolates	Kim et al., 2008
At5g57220	*cyp81F2*	cytochrome P450 monooxygenase	Pfalz et al., 2009
At3g09710	*iqd1*	IQ-Domain1	Levy et al., 2005
**LIPID METABOLISM**			
At3g01420	*α-dox1*	*α-dioxygenase1*	Avila et al., 2013
At3g11170	*fad7*	*fatty acid desaturase7*	Avila et al., 2012
At3g22400	*lox5*	*lipoxygenase 5* (9-lipoxygenase)	Nalam et al., 2012
At5g14180	*mpl1*	*Myzus persicae-induced lipase1*	Louis et al., 2010a
At2g43710	*ssi2*	*stearoyl-ACP desaturase*	Pegadaraju et al., 2005
**CARBOHYDRATE METABOLISM**			
At2g18700	*tps11*	*trehalose-6-phosphate synthase11*	Singh et al., 2011
At1g10550	*xth33*	*xyloglucan:xyloglucosyl transferase33*	Divol et al., 2007
**SENESCENCE AND OXIDATIVE BURST**			
At5g64930	*cpr5*	*constitutive expression of PR genes5*	Pegadaraju et al., 2005
At5g47910	*rbohd*	*respiratory burst oxidase homolog D*	Miller et al., 2009
**PHLOEM FUNCTION**			
At4g19840	*pp2-A1*	*phloem protein 2A1*	Zhang et al., 2011
**SMALL RNA GENE SILENCING PATHWAY**			
At1g01040	*dcl1*	*dicer-like1*	Kettles et al., 2013
At1g09700	*hyl1*	*hyponastic leaves 1*	Kettles et al., 2013
At4g20910	*hen1*	*hua enhancer1*	Kettles et al., 2013
At3g05040	*hst*	*hasty*	Kettles et al., 2013
At2g27100	*se*	*serrate*	Kettles et al., 2013
At1g48410	*ago1*	*argonaute1*	Kettles et al., 2013

a *CEV1 is involved in cellulose metabolism. JA and ethylene signaling are hyperactive in the cev1 mutant. JA signaling is required for the enhanced resistance phenotype of the cev1 mutant*.

b *Although PAD4 is associated with SA signaling and camalexin metabolism, PAD4's involvement in controlling GPA colonization is independent of SA signaling and camalexin metabolism*.

### Mechanisms that limit GPA infestation on arabidopsis

Both constitutive and inducible factors/mechanisms contribute to plant defense against aphids. In general, plant resistance mechanisms against aphids can be broadly classified as antixenosis and antibiosis (Painter, 1951; Kogan and Ortman, 1978). Antixenosis is used to describe mechanisms that result in a plant either not serving as a host, or given the option the insect preferring an alternate host. Antixenotic defenses could influence insect feeding behavior, for example adversely impacting its ability to find sieve elements. In contrast, antibiosis results from defenses that impact insect physiology leading to impairment of aphid growth, development, reproduction and/or survival (Smith, 2005). In some cases, antibiosis could also result from limited availability of nutrients required by an aphid (Pedigo, 1999). Severe cases of antibiosis could impact insect feeding behavior thus contributing to antixenosis, as well. Tolerance is another phenomenon that results in the plant withstanding or recovering from the infestation despite supporting an insect population that is comparable to that which causes damage on a susceptible variety (Painter, 1951). Thus, tolerance does not adversely impact the insect, but rather is an adaptation that benefits the plant. As discussed below, Arabidopsis engages both antibiotic and antixenotic defenses to control GPA infestation.

#### Perception of aphids

In plant-microbe interaction, immune receptors have been suggested to facilitate recognition of specific pathogen-derived effectors or infection-associated elicitors, leading to the activation of defenses that limit infection (Boller and Felix, 2009; Thomma et al., 2011; Gassmann and Bhattacharjee, 2012). Similar surveillance mechanisms likely allow plants to recognize aphid infestation (Smith and Clement, 2012). For example, in tomato the *Mi-1.2*-encoded nucleotide binding site (NBS) leucine-rich-repeat (LRR) protein confers resistance against certain biotypes of *Macrosiphum euphorbiae* (potato aphid) and *Meloidogyne* sp. (root-knot nematodes) (Milligan et al., 1998; Rossi et al., 1998; Vos et al., 1998), and in melon the *Vat*-encoded NBS-LRR protein confers resistance against *Aphis gossypii* (melon and cotton aphid) (Pauquet et al., 2004). Analogous to the involvement of immune receptors in plant immunity against pathogens, it is plausible that Mi-1.2 and Vat likely help recognize effectors delivered into the plant by the aphid or elicitors produced *in planta* in response to aphid infestation. Loci controlling resistance against aphids have been identified in other plants, as well. In lettuce resistance against *Nasonovia ribisnigri* (lettuce aphid) is conferred by the *Nr* gene, in soybean resistance against *Aphis glycines* (soybean aphid) is conferred by the *RAG1* and *RAG2* genes, in apple the *S*d_1_ gene confers resistance against some biotypes of *Dysaphis devecta* (rosy leaf curling aphid) (Roche et al., 1997), and ten loci (*Dn1-Dn9* and *Dnx*) have been identified in rye, wheat or Tausch's goatgrass that confer resistance against *Diuraphis noxia* (Russian wheat aphid) (Helden et al., 1993; Hill et al., 2006; Kim et al., 2010; Bouhssini et al., 2011; Smith and Clement, 2012).

Whether a similar immune receptor-mediated mechanism is utilized by plants against a generalist like the GPA is not known. However, a few recently concluded studies have demonstrated that GPA saliva contains factors that elicit defense responses in Arabidopsis. De Vos and Jander (2009) showed that saliva from the GPA when infiltrated into Arabidopsis leaves resulted in reduced GPA population size on the treated leaves. The resistance induced by saliva did not require salicylic acid (SA), jasmonic acid (JA) and ethylene signaling, which are involved in controlling infestation by insects and pathogens. The resistance inducing elicitor was sensitive to boiling and proteinase K treatment, suggesting that it is a protein. Size fractionation experiments indicated a 3–10 kD molecular range for this elicitor from GPA saliva. Expression levels of several Arabidopsis genes, including those associated with defense, signal transduction and senescence were altered in response to saliva application (De Vos and Jander, 2009), suggesting that the resistance enhancing effect of this proteinaceous elicitor is likely due to the activation of plant defense mechanisms. Mp10 and Mp42 are two salivary proteins from the GPA, which were recently shown to elicit host defenses and reduce insect fecundity when transiently over-expressed in *N. benthamiana* leaves (Bos et al., 2010). Mp10, which shows homology to insect protein olfactory segment D2-like protein (OS-D2-like protein), when over-expressed in *N. benthamiana* resulted in chlorosis. In addition Mp10 cross-talk with defense signaling mediated by the bacterial flg22 peptide resulted in the attenuation of flg22-induced ROS production (Bos et al., 2010). These results suggest that aphid salivary components, or products thereof, are likely recognized by Arabidopsis cells, leading to activation of defenses.

#### The tug-O-War for resources

Source-sink patterns are altered in aphid-infested plants resulting in the diversion of nutrient flow from the natural sinks to the aphid-infested organs (Mittler and Sylvester, 1961; Larson and Whitham, 1991; Dixon, 1998; Girousse et al., 2005). In Arabidopsis, GPA infestation resulted in changes in expression of genes involved in resource partitioning and sugar signaling (Moran and Thompson, 2001; Pegadaraju, 2005). GPA-infestation also resulted in increase in sucrose content in the aphid-infested organs (Singh et al., 2011). This increase in sucrose was relatively rapid, beginning as early as 6–12 h post infestation. Increase in sucrose content occurred even when the infested plants were kept in the dark, suggesting that changes in photosynthetic rate are not the likely cause of this increase. Increased turnover of other molecules could be a potential source for this increase in sucrose. Blockage in export of resources from the infested organ could also potentially contribute to this build-up of sucrose in the infested organ. Sucrose, which is responsible for the high osmolarity of the phloem sap is also the major feeding stimulant encountered by aphids while feeding on phloem sap (Douglas, 2006). However, aphids expend a lot of energy to counter the high osmolarity of the phloem sap they consume (Spiller et al., 1990; Pompon et al., 2010). For example, sucrases present in the insect gut hydrolyze sucrose to hexoses, which are then polymerized into oligosaccharides that have a lower contribution to osmotic pressure than hexoses and sucrose (Wilkinson et al., 1997; Ashford et al., 2000). Furthermore, these oligosaccharides are expelled out in the honeydew. Water consumption from the xylem has also been suggested as a means utilized by aphids to dilute the sugar content in the gut and thus contribute to osmoregulation (Spiller et al., 1990; Pompon et al., 2010). Thus, the infestation-associated increase in sucrose content in plant tissues could potentially be detrimental to the aphid. Genetic studies with the Arabidopsis *tps11* (*trehalose-6-phosphate synthase 11*) mutants suggest that this increase in sucrose in GPA-infested leaves is likely not detrimental to GPA. Quite to the contrary, GPA population was larger on the *tps11* mutant, despite sucrose content being 40% higher in the GPA-infested leaves of the *tps11* mutant compared to the wild type (WT) plants (Singh et al., 2011). Further studies are required to determine if this increase in sucrose in GPA-infested leaves is in the phloem sap consumed by the aphid, and/or is within the mesophyll cells of the infested leaves. In potato, antisense-mediated silencing of the *StSUT1* gene, which encodes a sucrose transporter, resulted in a reduction in sucrose content compared to the non-transgenic plants and simultaneously resulted in poor performance of potato aphid (Pescod et al., 2007). Taken together, these studies with *tps11* mutant and potato *StSUT1*-silenced lines suggest that aphids require an optimal level of sucrose and/or osmolarity and likely target sucrose accumulation to facilitate infestation.

GPA infestation also results in an increase in starch content in Arabidopsis leaves (Singh et al., 2011; Singh, 2012). This increase in starch was observed even when the plants were kept in complete darkness during the course of the experiment. Expression of the Arabidopsis *APL3* gene, which encodes a subunit of AGPase that synthesizes ADP-glucose, the donor of glucosyl moieties to the growing starch chain (Geigenberger, 2011), was also upregulated in GPA-infested plants (Singh, 2012). In Arabidopsis the *PGM1* gene encodes a phosphoglucomutase that synthesizes glucose-1-phosphate, which is the precursor for starch synthesis. *pgm1* mutants fail to accumulate starch (Yu et al., 2000). GPA population size was larger on the *pgm1* mutant compared to the WT plant (Singh et al., 2011; Singh, 2012). GPA numbers were also higher on a *gbss1* mutant that lacks a plastid-localized amylose synthesizing starch synthase activity (Singh, 2012). In contrast, GPA numbers were lower on the Arabidopsis *ssIII* (*starch synthase III*) mutant (Singh, 2012), which hyper-accumulates starch (Zhang et al., 2005) compared to the WT plant. Singh et al. (2011) have suggested that an increase in starch likely functions as a “secondary sink” that is associated with plant defense against the GPA, presumably by redirecting C into starch. Starch was previously shown to have an inhibitory effect on GPA feeding (Campbell et al., 1986). Hence, it is plausible that starch accumulation makes Arabidopsis leaves less desirable to the GPA.

Premature leaf senescence characterized by chlorophyll loss and upregulation of a subclass of *SENESCENCE ASSOCIATED GENES* (*SAG*) is observed in GPA-infested Arabidopsis (Pegadaraju et al., 2005; Louis et al., 2010b, 2012a). Expression of the *SAG* genes was induced within 24 h of infestation. As mentioned above, the GPA salivary protein Mp10 when expressed in leaves results in chlorosis (Bos et al., 2010). Thus, Mp10 is a likely elicitor of premature leaf senescence in GPA-infested Arabidopsis. Leaf senescence results in the export of nutrients from the senescing leaves and thus could potentially counter the ability of aphids to increase the sink strength of infested leaves. Indeed, transient expression of Mp10 in *N. benthamiana* also resulted in reduction of GPA fecundity (Bos et al., 2010). In support of a role for senescence in Arabidopsis defense, Pegadaraju et al. (2005) noted that the hyper-senescent *ssi2* and *cpr5* mutants exhibited enhanced resistance to GPA. Furthermore, mutation in the *PAD4* (*PHYTOALEXIN-DEFICIENT4*) gene, which resulted in the attenuation of the GPA infestation-induced premature leaf senescence phenotype, was accompanied by improved performance of the GPA. Senescence is accompanied by changes in redox status (Khanna-Chopra, 2012). Indeed, GPA infestation is accompanied by an increase in H_2_O_2_ content in potato leaves (Kerchev et al., 2012). Analysis of GPA performance on the Arabidopsis *rbohd* mutant, which is defective in ROS production, confirmed an important role for ROS in Arabidopsis defense against the GPA (Miller et al., 2009). The GPA attained a larger population size on the *rbohd* mutant than the WT plant (Miller et al., 2009). Thus, senescence associated physiological and developmental changes are presumably engaged by Arabidopsis to counter the ability of GPA to alter resource allocation and thereby control severity of aphid infestation. It is equally possible that senescence is accompanied by the production of factors that are toxic to the GPA.

#### Defenses in the phloem

***Phloem occlusion***. Phloem, the site of aphid feeding, provides an ideal location to pack defense metabolites as well as activate mechanisms that promote phloem occlusion (Walz et al., 2004; Will and van Bel, 2006; Gaupels et al., 2008). Comparison of insect feeding behavior with an Electrical Penetration Graph (EPG) set-up (Tjallingii, 1990; Tjallingii and Esch, 1993; Reese et al., 2000; Walker, 2000) demonstrated that Arabidopsis attempts to control GPA feeding from sieve elements (Pegadaraju et al., 2007; Singh et al., 2011). GPA spent more time feeding from the sieve elements on the *pad4* and *tps11* mutants than the WT plant (Pegadaraju et al., 2007; Singh et al., 2011). In contrast, time spent feeding from sieve elements was reduced when feeding on transgenic plants overexpressing *PAD4* (Pegadaraju et al., 2007), thus suggesting that *PAD4* and *TPS11*-mediated mechanisms are required for controlling GPA feeding from sieve elements. However, the involvement of SEO in *PAD4* and *TPS11*-dependent defense against GPA remains to be experimentally tested.

Callose deposition and phloem protein aggregation are mechanisms that contribute to SEO (Will and van Bel, 2006). Although the role of callose in Arabidopsis defense against GPA is not clear, studies with other plant-hemipteran interactions have suggested that callose is an important factor in defense against hemipteran insects. For example, callose deposition in rice is associated with resistance against the brown planthopper (*Nilaparvata lugens*) (Hao et al., 2008), and in Arabidopsis, expression of the *CALS1* gene, which encodes a callose synthase, was up-regulated and callose accumulation enhanced in response to *Bemisia tabaci* (silverleaf whitefly) infestation (Kempema et al., 2007).

Dispersion of forisomes, which appear to be composed of multiple SEO proteins, contributes to SEO in *Fabaceae*. In Arabidopsis, the *AtSEOR1* and *AtSEOR2* genes are homologs of the *Fabaceae* SEO protein-encoding genes (Anstead et al., 2012). However, loss of *AtSEOR1* or *AtSEOR2* function in the *atseor1* and *atseor2* mutants, respectively, did not adversely impact basal resistance against GPA (Anstead et al., 2012). Quite to the contrary, the number of nymphs produced was higher on the WT than the *atseor1* and *atseor2* mutant plants, suggesting that presence of these proteins is beneficial to the insect. Forisome dispersion also was not observed in faba bean (*Vicia faba*) in response to penetration of sieve elements by stylets of *Acyrthosiphon pisum* (pea aphid) (Walker and Medina-Ortega, 2012). However, when forisome dispersal was induced in *Megoura viciae* (vetch aphid)-infested faba bean leaves by burning of leaf-tips, changes were observed in insect feeding behavior. EPG analysis showed that the insect switched from phloem sap ingestion to secretion of water saliva, presumably to reverse phloem occlusion (Will et al., 2007). A transition from phloem-sap consumption to salivation has also been observed in similar experiments conducted with several other plants and aphids (Will et al., 2009), thus suggesting that this behavioral response is likely a general response of aphids to phloem occlusion. Will et al. (2007) showed that watery saliva from vetch aphid was capable of reversing forisome dispersal *in vitro*, thus suggesting that aphid saliva contains factors that have the ability to reverse phloem occlusion mediated by forisome dispersion.

***Antibiotic factors in the phloem***. The phloem sap of Arabidopsis contains an antibiotic factor that is detrimental to GPA. Phloem sap-enriched petiole exudates collected from leaves of uninfested Arabidopsis plants when added to synthetic diet lowered GPA fecundity (Louis et al., 2010a,b, 2012a; Singh et al., 2011; Nalam et al., 2012). The Arabidopsis *ssi2* mutant, which exhibits heightened resistance to GPA, contains elevated levels of this antibiosis activity (Louis et al., 2010a,b). The Arabidopsis *PAD4* and *MYZUS PERSICAE-INDUCED LIPASE 1* (*MPL1*) genes were required for the increased antibiosis observed in the *ssi2* mutant (Louis et al., 2010a,b). In agreement with a role for this *PAD4*- and *MPL1*-dependent antibiotic activites in controlling overall severity of GPA infestation, the *ssi2*-depdendent antibiosis activity was lower in *ssi2 pad4* and *ssi2 mpl1* double mutant plants. Furthermore, petiole exudates from uninfested leaves of *pad4* and *mpl1* contained lower levels of this antibiotic activity (Louis et al., 2010a,b). The identity of this antibiotic factor that is altered in the *ssi2*, *pad4*, and *mpl1* mutants is not known. As discussed below, Arabidopsis phloem sap contains proteins and non-protein metabolites that are detrimental to GPA. Accumulation of one or more of these factors could potentially be dependent on PAD4 and/or MPL1 activity. Despite the presence of these detrimental factors in the phloem sap, GPA is capable of colonizing Arabidopsis. This is in part because GPA adapts on Arabidopsis to suppress the accumulation and/or detoxify one or more of these factors. As discussed later, oxylipins produced by the host facilitate adaptation of GPA on the host plant allowing it to suppress accumulation of this antibiosis activity (Nalam et al., 2012).

Plants in the Brassicaceae family, which includes Arabidopsis, contain glucosinolates, which are defensive compounds. Glucosinolate accumulation is under control of JA signaling (Mewis et al., 2005), which as discussed below has been suggested to promote resistance against GPA. The metabolism of glucosinolates and their role in plant defense has been reviewed recently (Hopkins et al., 2009; Sønderby et al., 2010; Wittstock and Burow, 2010; Winde and Wittstock, 2011). When acted upon by myrosinases, glucosinolates can release toxic breakdown products that are detrimental to insects (Chew, 1988; Louda and Mole, 1991; Rask et al., 2000). The accumulation of glucosinolates and myrosinases is compartmentalized, thus preventing their mixing in the absence of physical damage to the plant tissue. Glucosinolates are stored in the sulfur-rich S-cells that are in close proximity to the phloem (Koroleva et al., 2000). Myrosinases on the other hand are stored in the myrosin cells and guard cells (Andréasson and Jørgensen, 2003; Zhao et al., 2008). In Arabidopsis, GPA infestation results in elevated levels of glucosinolates that can also be detected in the phloem sap (Kim and Jander, 2007; Louis et al., 2010a). Expression of genes putatively involved in glucosinolate metabolism were also upregulated in Arabidopsis leaves infested with the GPA and in leaves treated with saliva from the GPA (Mewis et al., 2006; De Vos and Jander, 2009). A defensive role for glucosinolates was suggested by the observation that resistance to the GPA was increased in the Arabidopsis *atr1D* mutant, which accumulates higher amounts of indole-glucosinolates than the WT plant (Kim et al., 2008). By contrast, in comparison to the WT plant, the *cyp79B2 cyp79B3* double mutant that does not accumulate indole-glucosinolates exhibited enhanced susceptibility to the GPA. Mutations in the *CYP81F2* gene, which encodes a cytochrome P450 monooxygenae that is required for synthesis of 4-hydroxy-indole-3-yl-methyl glucosinolate also resulted in lowered resistance to GPA (Pfalz et al., 2009). The feeding style of aphids, which causes minimal physical damage to cells surrounding the phloem, limits the mixing of glucosinolates and myrosinases, thus allowing intact glucosinolates to be ingested from the phloem by the aphid. Indeed, glucosinolates have been detected in the honeydew of GPA reared on Arabidopsis (Kim and Jander, 2007). However, the indole class of glucosinolates were metabolized in the insect gut (Kim et al., 2008) and the products were found to have a negative effect of GPA settling and growth (Kim and Jander, 2007; Kim et al., 2008; Pfalz et al., 2009). Glucosinolate level and composition was not significantly impacted in the *pad4* and *mpl1* mutants, suggesting that their accumulation is not under control of *PAD4* and *MPL1* (Kim and Jander, 2007; Louis et al., 2010a).

Lectins, also known as agglutinins, are proteins that can reversibly and specifically bind to carbohydrates. Many lectins are toxic to phytophagous insects (Vandenborre et al., 2011). The Arabidopsis Phloem Protein2-A1 (PP2-A1), which is a component of phloem protein bodies, is also a lectin. When added to a synthetic diet, recombinant PP2-A1 affected weight gain in GPA and soybean aphid nymphs (Beneteau et al., 2010). Lectins have an affinity for carbohydrates, which may interfere with physiological processes in the insect gut, thus controlling insect infestation (Carlini and Grossi-de-Sa, 2002; Vasconcelos and Oliveira, 2004). Confirming a role for PP2-A1 in Arabidopsis defense against GPA, constitutive expression of PP2-A1 adversely impacted the ability of GPA to feed from the sieve elements (Zhang et al., 2011). The *PP2-A1* gene was also required for harpin to promote resistance against GPA (Zhang et al., 2011). Harpin is a hypersensitive response-inducing protein produced by gram negative pathogenic bacteria that also elicits defenses against pathogens.

*N*^δ^-acetylornithine is a novel class of non-protein amino acid that was identified in the phloem sap of methyl-JA-treated Arabidopsis (Adio et al., 2011). GPA infested plants contained elevated levels of *N*^δ^-acetylornithine. Furthermore, GPA reproduction was significantly reduced when the aphids were fed on a diet containing *N*^δ^-acetylornithine, suggesting that this non-protein amino acid has a defensive role against aphids (Adio et al., 2011). The toxic effect of this compound is specific to phloem-feeding insects, since it did not have any effect on the growth of lepidopteran caterpillars. Expression of the *NATA1* gene, which is involved in the biosynthesis of *N*^δ^-acetylornithine in Arabidopsis, is induced upon GPA infestation. Furthermore, its expression is high in vascular tissues, thus bolstering the notion that this aphidicidal compound is synthesized and/or accumulates in the phloem (Adio et al., 2011).

#### Contribution of arabidopsis lipid metabolism to defense against GPA

Besides functioning as major structural components of cell membranes, plant lipids also function as precursors of antibiotic compounds and signaling molecules (Wang, 2004; Shah, 2005; Wasternack, 2007; Upchurch, 2008; Scherer, 2010; Yan et al., 2013). In addition, lipids have also been implicated in cross-kingdom communication (Christensen and Kolomiets, 2011). Lipid metabolism in Arabidopsis also impacts interaction with the GPA. A loss-of-function mutation in the *SSI2* gene, which encodes the major plastidyl stearoyl acyl-carrier protein desaturase that catalyzes the synthesis of oleic acid in Arabidopsis, resulted in enhanced resistance against the GPA (Louis et al., 2010b). As mentioned above, the *ssi2* mutant exhibits a hyper-senescence phenotype that is characterized by constitutively elevated expression of the *SAG13* gene and spontaneous cell death. Furthermore, petiole exudates from *ssi2* accumulate elevated levels of antibiosis activity against the GPA. EPG analysis indicated that the GPA feeding behavior was not adversely impacted on the *ssi2* mutant compared to the WT plant. The *ssi2* mutant also accumulates elevated levels of SA. Since, SA added to a synthetic diet had an adverse impact on insect fecundity, Louis et al. (2010b) conducted experiments with the *ssi2 nahG* plants, in which the *ssi2*-dependent accumulation of SA is attenuated, to determine if SA is indeed required for the *ssi2*-conferred enhanced resistance against GPA. Although presence of *nahG* had a weak effect on the strength of antibiosis activity in petiole exudates, this was not sufficient to weaken the *ssi2*-conferred resistance against GPA, thus indicating that SA is not a major factor contributing to the *ssi2*-conferred enhanced resistance (Louis et al., 2010b).

Oxylipins (oxidized lipids) contribute to Arabidopsis defense to the GPA. Oxidation of polyunsaturated fatty acid (e.g., linoleic and linolenic acids) is the first step in the synthesis of oxylipins (Feussner and Wasternack, 2002; Mosblech et al., 2009). Enzymatic oxidation of polyunsaturated fatty acids is mediated by enzymes like lipoxygenases (LOXs) and α-dioxygenases (α-DOXs). The resultant oxidized fatty acids can be further processed enzymatically or non-enzymatically to yield a variety of oxylipins, several of which have roles in plant stress response. JA is one of the best studied oxylipin that is derived via the LOX pathway. Its role in Arabidopsis-GPA interaction is discussed later. Oxidation of fatty acids by α-DOXs yields 2(R)-hydroperoxides, which can be further processed into other products. In Arabidopsis, expression of the α-*DOX1* gene was up-regulated in response to GPA infestation (Avila et al., 2013). Similarly, in tomato expression of the *Sl*α-*DOX1* gene was up-regulated in response to potato aphid infestation (Avila et al., 2013). In both, Arabidopsis and tomato, knock-down of α-DOX1 function resulted in increased susceptibility to aphids. In comparison to the WT plant, GPA population size was larger on the Arabidopsis α-*dox1* mutant (Avila et al., 2013). Similarly, virus-induced gene silencing of *Sl*α-*DOX1* in tomato resulted in an increase in the size of potato aphid population compared to the non-silenced plants (Avila et al., 2013). α-DOX1-derived lipids have known antibiotic and signaling functions, either or both of which could contribute to the α-*DOX1*-mediated resistance against aphids. As discussed later, recent studies indicate that oxylipins also are susceptibility factors in Arabidopsis interaction with GPA.

*MPL1*, which is required for the *ssi2*-dependent enhanced resistance and hyper-accumulation of antibiosis activity in petiole exudates, encodes a protein with homology to α/β-fold acyl hydrolases/lipases (Louis et al., 2010a). A signal peptide at the N-terminus suggests that MPL1 is likely targeted to the endoplasmic reticulum. MPL1 contains a signature GXSXG esterase/lipase catalytic motif and recombinant MPL1 possesses lipase activity. *MPL1* expression was induced in response to GPA infestation and petiole exudates from the *mpl1* mutant contained lower levels of antibiosis activity compared to WT plant (Louis et al., 2010a). In contrast, MPL1 overexpression resulted in an increase in antibiosis activity, which was paralleled by an increase in resistance against GPA (Louis et al., 2010a). Loss of MPL1 function had no impact on antixenosis against GPA. The feeding behavior of GPA was comparable when feeding on WT and *mpl1* mutant (Louis et al., 2010a). Although the exact identity of the MPL1-dependent antibiotic factor is not known, it could possibly be a lipid/lipid-derived product present in the phloem sap. The phloem sap is known to contain a variety of lipids including oxylipins (Benning et al., 2012). One or more products of MPL1 activity could be directly toxic to the insect. Alternatively, an MPL1-derived lipid might indirectly impact GPA infestation. MPL1 also contains a HX_4_D acyltransferase motif suggesting that it could be involved in lipid modifications. Whether the lipase activity or the acyltransferase motif of MPL1 are required for its involvement in Arabidopsis-GPA interaction remains to be determined.

### Regulation of defenses

#### Role of salicylic acid, jasmonic acid and ethylene in arabidopsis-GPA interaction

SA and JA have important signaling functions in plant defense against pathogens. In some cases of plant-pathogen interaction they function together to promote resistance, while in other cases SA and JA have an antagonistic relationship (Mur et al., 2006; Pieterse et al., 2009; Robert-Seilaniantz et al., 2011). SA and JA signaling also have a role in some cases of plant defense against aphids. In tomato, SA signaling is required for *Mi-1.2*-mediated resistance against the potato aphid (Li et al., 2006). Furthermore, gene expression studies conducted in Arabidopsis indicate that GPA infestation triggers the induction of the SA and JA pathway. For example, expression of the *PATHOGENESIS-RELATED 1* (*PR1*) gene, which for long has been used as a molecular marker for the activation of SA signaling, and the *PDF1.2* gene, which is a marker for the activation of JA and ethylene signaling, were upregulated in GPA-infested leaves (Moran and Thompson, 2001; Moran et al., 2002; De Vos et al., 2005; Pegadaraju, 2005; Mewis et al., 2006). Similarly in tomato, induction of the SA-inducible *P4* gene, which is homologous to the Arabidopsis *PR1* gene, has also been noted in plants infested with the potato aphid (Li et al., 2006). Expression of the isochorismate synthase encoding *ICS1* (*SID2*) gene, which is involved in the synthesis of SA in Arabidopsis, was also upregulated in response to GPA infestation (Pegadaraju, 2005). Similarly, expression of *ENHANCED DISEASE SUSCEPTIBILITY 5* (*EDS5*), which is required for SA synthesis, was also induced in GPA-infested plants (Pegadaraju, 2005). However, De Vos et al. (2005) reported that GPA infestation did not result in any observable increase in SA content in Arabidopsis. As suggested by them, this inability to see an increase in SA could be due to fewer cells responding to GPA infestation as opposed to that seen in pathogen-infected tissues. Genetic studies conducted by several groups have indicated that although GPA infestation activates SA signaling, SA signaling is not important for promoting resistance against GPA. Mutations in the *ICS1* and *EDS5* genes did not result in increased colonization by GPA on the *ics1* and *eds5* mutants, compared to WT plants (Moran and Thompson, 2001; Pegadaraju et al., 2005). Furthermore, Pegadaraju et al. (2005) reported that GPA population size was comparable between WT and transgenic Arabidopsis plants expressing the bacterial *nahG*-encoded salicylate hydroxylase, which converts SA to catechol. Loss of NPR1 (NON-EXPRESSER OF PR GENES1), a key SA signaling regulator and a putative SA receptor (Wu et al., 2012), also did not result in improved performance of GPA on the *npr1* mutant than the WT plant (Moran and Thompson, 2001; Mewis et al., 2005; Pegadaraju et al., 2005). Finally, application of (1, 2, 3) thiadiazole-7-carbothioic acid (*S*) methyl ester (BTH), a synthetic functional analog of SA, also did not curtail GPA colonization on Arabidopsis (Moran and Thompson, 2001). Taken together, these results confirm that SA signaling is not critical for controlling GPA infestation on Arabidopsis. Quite to the contrary, Mewis et al. (2005) reported that GPA population size was smaller on the NahG and *npr1* plants than on the WT plant, suggesting that SA signaling in fact might be promoting susceptibility to GPA.

According to a “decoy” hypothesis involving SA, some insects may have evolved to trick the host into activating SA signaling, which in many cases is known to antagonize the activation of JA signaling (Walling, 2008). In support for a potential function of the JA pathway in promoting resistance against GPA, Ellis et al. (2002a) reported that the *cev1* (*constitutive expression of VSP1*) mutant, which contains higher levels of JA than the WT plant, was more resistant to GPA than the WT plant. They further showed that exogenously applied MeJA also promoted resistance to GPA in Arabidopsis (Ellis et al., 2002a). In contrast, GPA numbers were higher on JA-insensitive *coi1* (*coronatine insensitive1*) mutant compared to the WT plant (Ellis et al., 2002a; Mewis et al., 2005, 2006). JA is known to promote the accumulation of metabolites like indole-glucosinolates, camalexin and the non-protein amino acid *N*^δ^-acetylornithine (Zhou et al., 1999; Mikkelsen et al., 2003; Adio et al., 2011) that are detrimental to GPA. However, the simultaneous deficiency of ω3 fatty acid desaturases encoded by the Arabidopsis *FAD7* and *FAD8* genes, which are required for the synthesis of trienoic fatty acids, the precursors for JA synthesis, resulted in enhanced resistance to GPA (Avila et al., 2012). Similar to the results with the Arabidopsis *fad7 fad8* double mutant, in tomato, loss of *FAD7* activity in the *spr2* mutant resulted in enhanced resistance against the potato aphid (Avila et al., 2012). This increase in resistance in *spr2* was not associated with JA signaling, but instead resulted from elevated content of SA and increased *Sl*α-*DOX1* gene activity in the *spr2* mutant (Avila et al., 2012, 2013). Mutations in other genes involved in JA synthesis also did not influence potato aphid colonization on tomato (Avila et al., 2012), although previous studies indicated that MeJA application enhanced resistance against potato aphid in tomato (Cooper et al., 2004). Considering that *COI1* is a component of the proteasomal protein turnover mechanism that likely also impacts processes other than JA (He et al., 2012; Ralhan et al., 2012), and Arabidopsis CEV1 is a subunit of cellulose synthase, the lack of which has pleiotropic effects in the *cev1* mutant that include the simultaneous hyperactivation of ethylene and JA signaling (Ellis et al., 2002b), the results with the *fad7 fad8* mutant (Avila et al., 2012), in conjunction with a previous report by De Vos et al. (2005), who failed to see any significant increase in JA in GPA-infested Arabidopsis, call for a careful reevaluation of JA's role in Arabidopsis defense against GPA.

Ethylene production is elevated in aphid infested plants. For example, increases in ethylene production was observed in tomato infested with potato aphid, alfalfa infested with *Therioaphis maculate* (spotted alfalfa aphid), wheat infested with *Schizaphis graminum* (greenbug), and barley infested with *Rhopalosiphum padi* (oat aphid) and Russian wheat aphid (Dillwith et al., 1991; Anderson and Peters, 1994; Miller et al., 1994; Argandoña et al., 2001; Mantelin et al., 2009). Changes in expression of genes putatively involved in ethylene metabolism/signaling also have been observed in melon plants responding to the melon and cotton aphid infestation, in tomato infested with the potato aphid, and Arabidopsis infested with the GPA (Moran et al., 2002; Anstead et al., 2010; Mantelin et al., 2009). In melon, the expression of ethylene pathway and response genes was reported to be highly upregulated during the resistant interaction mediated by the *Vat* resistance gene, thus leading the authors to suggest that ethylene may have a role in *Vat*-mediated resistance (Anstead et al., 2010). Quite to the contrary, genetic studies in tomato indicated that ethylene signaling contributes to susceptibility to the potato aphid in the absence of the *Mi-1.2* resistance gene function (Mantelin et al., 2009). In choice test assays, the potato aphid was observed to prefer the WT plant to the ethylene-insensitive *Never ripe* mutant. Similarly, in Arabidopsis, Mewis et al. (2006) reported that GPA and cabbage aphid populations were significantly smaller on the ethylene-insensitive *etr1* mutant than the corresponding WT plant, thus suggesting that like in the tomato-potato aphid interaction, ethylene likely contributes to Arabidopsis susceptibility to the GPA and the cabbage aphid. However, in another study the same group did not observe significant differences in GPA population size on the *etr1* mutant compared to WT plants (Mewis et al., 2005). Similarly, Kettles et al. (2013) did not observe significant differences in GPA fecundity on insects reared on WT and *etr1* mutant. However, a higher fecundity was observed for GPA reared on the ethylene-insensitive *ein2* mutant than the WT plant (Kettles et al., 2013).

Ethylene signaling was also required in Arabidopsis for harpin-induced resistance against GPA (Dong et al., 2004). Harpin treatment limited insect settlement, feeding and fecundity on Arabidopsis (Dong et al., 2004; Liu et al., 2010; Zhang et al., 2011). Unlike the WT plants, harpin was unable to limit insect fecundity on the *etr1* and *ein2* mutants (Dong et al., 2004). Liu et al. (2010) further demonstrated that the ethylene and harpin up-regulated the *AtMYB44* gene, which encodes a Myb-family transcription factor, was required for harpin-induced resistance to GPA. *AtMYB44* expression is also induced in response to GPA infestation and application of GPA saliva (De Vos and Jander, 2009). However, whether *AtMYB44* is required for basal resistance against GPA was not reported. *AtMYB44* was required for the harpin-induced up-regulation of *EIN2* expression. Also required for harpin-induced resistance against GPA, but not for *EIN2* induction, were *AtMYB15*, *AtMYB51*, and *AtMYB73*, three other Myb-family transcription factor encoding genes, which are also ethylene inducible (Liu et al., 2010). Ethylene and harpin also induced expression of *AtMYB30*, *AtMYB108*, *AtZFP6*, and *AtRAP2.6L*. However, mutations in these genes enhanced harpin-induced resistance to GPA, suggesting that these genes might be involved in dampening of harpin- and likely ethylene-induced defense signaling. Ethylene-signaling thus may be involved in promoting expression of both, genes that promote resistance as well as those that promote susceptibility to GPA. This might explain the discrepancies observed between various studies (Dong et al., 2004; Mewis et al., 2005, 2006; Kettles et al., 2013) on the impact of ethylene-insensitive mutants on Arabidopsis-GPA interaction.

#### PAD4, a regulator of antixenosis, antibiosis and premature leaf senescence in GPA-infested arabidopsis

The Arabidopsis *PAD4* gene, which encodes a nucleocytoplasmic protein, is required for defense against a variety of pathogens and the GPA (Weirmer et al., 2005; Louis et al., 2012a). *PAD4* was found to be required for modulating SA accumulation in pathogen-infected plants (Zhou et al., 1998; Jirage et al., 1999; Rietz et al., 2011), and for systemic acquired resistance, an inducible defense mechanism that increases resistance against subsequent infections in plants that previously experienced a localized infection (Shah and Zeier, 2013). As mentioned above, in Arabidopsis-GPA interaction, *PAD4* function is required for antibiosis and antixenosis. When given a choice between the WT and the *pad4* mutant, GPA preferred to settle on the *pad4* mutant (Pegadaraju et al., 2007). In contrast, when given a choice between the WT and plants constitutively expressing *PAD4* from the *CaMV 35S* promoter, GPA preferred the WT plant, thus suggesting that *PAD4* is required for the accumulation of a factor that deters insect settling on Arabidopsis. EPG analysis indicated that compared to GPA feeding on the WT plant, insects on the *pad4* mutant spent more time in the sieve element phase (Pegadaraju et al., 2007), suggesting that the *pad4* mutant lacked a factor that controls insect feeding from sieve elements. In contrast, on plants constitutively expressing *PAD4*, GPA spent less time in the sieve element phase and the plants exhibited enhanced resistance to GPA (Pegadaraju et al., 2007). Petiole exudates collected from the *pad4* mutant lacked the antibiosis activity that is present in petiole exudates collected from WT plants (Louis et al., 2010b). Analysis of plants expressing the coding sequence of the GUS reporter from the *PAD4* promoter indicated that the *PAD4* promoter directs GUS expression in cells at the feeding site (Louis et al., 2012b). Thus, the feeding deterrence function of *PAD4* is likely due to its function in the phloem or cells surrounding the phloem. Glucosinolate content was not affected in the *pad4* mutant (Kim and Jander, 2007), thus suggesting that the PAD4-mediated resistance likely does not involve glucosinolates.

*PAD4* is also required for the timely activation of leaf senescence in GPA-infested Arabidopsis. Senescence associated chlorophyll loss was slower in the *pad4* mutant than the WT plant (Pegadaraju et al., 2005). Expression of the *SAG* genes was induced slower in the *pad4* mutant (Pegadaraju et al., 2005; Louis et al., 2010b, 2012a). Although overexpression of *PAD4* did not constitutively activate *SAG* gene expression, GPA infestation resulted in the faster activation of *SAG* genes (Pegadaraju et al., 2007). The fact that mere expression of *PAD4* was not sufficient for the induction of leaf senescence, suggests that additional factors are required for promoting leaf senescence in response to aphid infestation.

PAD4 exhibits homology to acyl hydrolases/esterases/lipases. The N-terminal half of PAD4 contains a triad of amino acids (S118, D178, and H229) (Jirage et al., 1999; Feys et al., 2005), which in other acyl hydrolases/lipases are part of the catalytic triad (Blow, 1990). However, no hydrolytic activity has been demonstrated for PAD4. On plants expressing missense versions of PAD4 (*PAD4*[S118A] and *PAD4*[D178A]) GPA population size was larger than on plants expressing WT PAD4, thus suggesting that S118 and D178 are critical for PAD4's involvement in defense against GPA (Louis et al., 2012a). However, the *PAD4*[S118A] allele did not impact plant choice by GPA. Furthermore, the PAD4-modulated expression of *SAGs* in response to GPA infestation also was not adversely impacted by this mutant alleles. Feeding behavior analysis with the EPG technique revealed that insects spent more time feeding from the sieve elements of plants expressing the *PAD4*[S118A] allele (Louis et al., 2012a). In addition, petiole exudates from plants expressing *PAD4*[S118A] were also deficient in the *PAD4*-dependent antibiosis activity. These results suggest that S118 in PAD4 is required for its antibiosis function and for deterring feeding from sieve elements. Thus, two distinct PAD4 activities contribute to defenses against GPA. The first activity, which limits insect feeding and promotes antibiosis, is dependent on S118. The second activity, which deters insect settling and promotes *PAD4* expression, does not require S118.

In pathogen-infected Arabidopsis, PAD4 functioning along with its interacting protein EDS1 (ENHANCED DISEASE SUSCEPTIBILITY 1), regulates its own expression and functions in a feed-positive SA amplification loop with EDS1 to regulate expression of defense genes (Weirmer et al., 2005). *EDS1* expression is induced in GPA-infested plants (Pegadaraju et al., 2007). However, *EDS1* was not required for controlling GPA infestation (Pegadaraju et al., 2007; Louis et al., 2012a,b). GPA population size was comparable between the WT and the *eds1* mutant. As mentioned above, SA is also not critical for controlling GPA infestation. Taken together, these studies indicate that PAD4-mediated defense against GPA is unique in that it does not invoke EDS1 and SA. It is plausible that the two different PAD4 molecular activities that are required for controlling GPA infestation involve PAD4 interaction with separate protein(s).

*PAD4* was identified by Glazebrook et al. (1997) in a screen for Arabidopsis mutants that were deficient in the accumulation of the phytoalexin, camalexin. *PAD4* regulates expression of *PAD3*, which encodes a P450 monooxygenase involved in camalexin biosynthesis (Zhou et al., 1999). Expression of *PAD3* was up-regulated in GPA-infested Arabidopsis leaves as well as in response to GPA saliva (Pegadaraju et al., 2005; De Vos and Jander, 2009). Furthermore, camalexin levels were found to increase in response to GPA infestation (Louis, 2011; Kettles et al., 2013). However, *PAD4* function was not required for this increase in camalexin in GPA-infested Arabidopsis (Louis, 2011). Furthermore, Pegadaraju et al. (2005) observed that GPA population size was comparable between the WT and the *pad3* mutant, thus leading them to conclude that camalexin is not important for the *PAD4*-mediated resistance against GPA. In their bioassays with GPA on Arabidopsis, Pegadaraju et al. (2005) used insects that were reared on a mix of mustard and radish plants. Furthermore, the bioassays were conducted over a 2 day period. In contrast to the observations of Pegadaraju et al. (2005), Kettles et al. (2013) observed that in a different experimental set-up involving 2 week-long fecundity assays, GPA nymphs that were born on the *pad3* mutant and reared to adulthood on the *pad3* mutant exhibited higher fecundity than nymphs born and raised on WT plants. Furthermore, they observed that camalexin provided in an artificial diet had a detrimental effect on GPA fecundity. Taken together, these two studies (Pegadaraju et al., 2005; Kettles et al., 2013) suggest that the role of *PAD3* and camalexin in defense against GPA might be influenced by the length of time the insects are reared on Arabidopsis. *PAD3* and camalexin synthesis are also required for controlling cabbage aphid infestation on Arabidopsis (Kuśnierczyk et al., 2008).

#### TPS11-dependent trehalose metabolism regulates defense against GPA

The non-reducing α,α-1,1-linked glucose disaccharide trehalose, which serves as an energy source and an osmoprotectant in lower organisms, has a role in the protection of plants from stress (Schluepmann et al., 2003; Paul et al., 2008; Fernandez et al., 2010). Trehalose-6-phosphate (T6P) and trehalose are suggested to have signaling function in plants (Paul et al., 2008; Fernandez et al., 2010). In plants, trehalose is synthesized in two steps. The first step involves the synthesis of T6P by T6P synthase. In the subsequent step, T6P is dephosphorylated by T6P phosphatase to yield trehalose. Recently, it was suggested that trehalose or a derivative has a novel signaling function in plant defense against GPA (Singh et al., 2011; Hodge et al., 2012). Trehalose levels increased in response to GPA infestation. This increase in trehalose accumulation in GPA-infested plants was paralleled by a transient elevation of *TPS11* expression (Singh et al., 2011). *TPS11* encodes a protein with T6P synthase and T6P phosphatase activities. The GPA infestation-induced accumulation of trehalose was attenuated in the *tps11* mutant, allowing increased colonization of the mutant by GPA. In contrast, resistance was enhanced in *tre1* (*trehalase*) mutant, which accumulates elevated levels of trehalose due to deficiency in the ability to degrade trehalose (Singh et al., 2011). Similarly, trehalose content and resistance to GPA were higher in transgenic *otsB* plants, which overexpress the T6P phosphatase-encoding bacterial *otsB* coding sequence. Like the *pad4* mutant, the *tps11* mutant was preferred by GPA over WT plants and petiole exudates of the *tps11* plants contained reduced antibiosis activity. EPG characterization of GPA feeding behavior indicated that GPA spent more time in the sieve element phase on the *tps11* mutant than the WT plant. Trehalose application restored resistance to the *tps11* mutant, thus confirming that trehalose deficiency was cause of the *tps11* phenotypes.

Trehalose application induces *PAD4* expression, which was constitutively higher in the trehalose hyper-accumulating *otsB* plant as well as *TPS11* overexpressing plants (Singh et al., 2011). GPA infestation-induced expression of *PAD4* was delayed in the *tps11* mutant compared to the WT plant, thus indicating that *TPS11*-dependent trehalose increases are required for the timing and strength of *PAD4* expression in GPA-infested Arabidopsis (Singh et al., 2011). *TPS11* also influences starch accumulation in GPA-infested plants. Starch content was lower in the GPA-infested *tps11* mutant compared to the WT plant (Singh et al., 2011). In comparison, sucrose content was higher on the *tps11* mutant. These results suggest that *TPS11*-dependent trehalose, promotes the flux of C from sucrose into starch, thereby contributing to overall defense against GPA. As mentioned above, starch accumulation is a likely physiological adaptation that helps the plant to counter the GPA.

The phloem sap of GPA-infested Arabidopsis contains elevated levels of trehalose (Hodge et al., 2012), thus suggesting that trehalose can potentially act as a phloem-mobile signal. Hodge et al. (2012) further observed that trehalose content in GPA was higher when cultivated on Arabidopsis than on synthetic medium or on other plants that did not accumulate trehalose in response to GPA infestation, leading them to suggest that when on Arabidopsis the insect assimilates trehalose from phloem sap. Although trehalose is a major sugar in the insect hemolymph, when added to a synthetic diet trehalose was detrimental to GPA (Singh et al., 2011), thus suggesting that *de novo* synthesized trehalose in the hemolymph might have a different physiological effect than trehalose in the gut. Thus, trehalose consumed by the insect when feeding on Arabidopsis could potentially be detrimental to the insect. The mid-gut of pea aphid and soybean aphid contain trehalase (Cristofoletti et al., 2003; Bansal et al., 2013), suggesting that some aphids likely utilize trehalase in the gut to counter the presence of trehalose consumed from plants. Thus, trehalose might have a dual role in plant-aphid interaction, functioning as a signaling molecule to promote plant defenses and simultaneously having a detrimental effect on insect physiology.

GPA infestation also promoted trehalose accumulation in leaves of tomato plants (Singh and Shah, 2012). Expression of the *TPS11* and *PAD4* homologs were up-regulated in GPA-infested tomato, which also accumulated elevated levels of sucrose and starch. Furthermore, trehalose application induced expression of the tomato *PAD4* homolog, promoted starch accumulation and limited insect colonization, thus suggesting that the role of trehalose in defense against GPA is likely conserved in other species, as well. Hodge et al. (2012) however, did not detect trehalose accumulation in GPA infested spring cabbage and black mustard as compared to Arabidopsis, suggesting that the interaction between the GPA and additional hosts needs to be studies in order to determine the extent to which the engagement of trehalose metabolism in plant defense against aphids has been conserved.

Arabidopsis contains more than 20 genes putatively involved in trehalose metabolism (Rolland et al., 2006; Paul et al., 2008). Whether the trehalose that accumulates in response to GPA infestation in Arabidopsis leaves is a product of *TPS11* activity, or is synthesized by one or more of the other enzymes, is not known. It is plausible that TPS11-synthesized T6P and/or trehalose functions as a signal to activate expression and/or activity of one or more of the other trehalose biosynthesis genes/enzymes, which contribute to the overall increase in trehalose in GPA-infested plants. *TPS11* expression is also altered in plants exposed to other abiotic and biotic stressors (Golem and Culver, 2003; Suzuki et al., 2005; Fujita et al., 2007; Iordachescu and Imai, 2008), suggesting that the *TPS11*-regulated mechanism(s) has a wider role in plant stress response.

#### Role of small RNAs in arabidopsis-GPA interaction

Small RNAs, which are 20–40 nucleotide-long non-coding RNAs, regulate gene expression at the transcriptional and post-transcriptional level. In plants, small RNAs are known to regulate JA and ethylene signaling, and influence defense against microbes and herbivores (Pandey and Baldwin, 2007; Pandey et al., 2008; Katiyar-Agarwal and Jin, 2010). Genetic studies in Arabidopsis indicated that mutations in plant microRNA (miRNA) pathway significantly impacted GPA fecundity (Kettles et al., 2013). GPA fecundity was lower on plants that contained mutations in the *DCL1* (*DICER-LIKE1*), *HYL1* (*HYPONASTIC LEAVES 1*), *HEN1* (*HUA ENHANCER1*), *SE* (*SERRATE*), and *HST* (*HASTY*) genes, which are involved in miRNA generation, processing or transport, and in the *AGO1* (*ARGONAUTE1*) gene, which is one of the slicer in the miRNA pathway. Expression of the *CYP83B1* and *CYP81F2* genes, which are involved in the synthesis of indolic glucosinolates, and the *CYP79B2* gene, which is involved in the synthesis of indole-3-acetaldoxime, a precursor for indolic glucosinolates as well as auxin and camalexins, was constitutively higher in the *dcl1* mutant. In addition, *PAD3* induction and camalexin accumulation in response to GPA infestation were higher in the *dcl1* mutant than the WT plant. GPA fecundity was significantly higher on the *dcl1 pad3* double mutant compared to the *dcl1* mutant plant, thus suggesting that increased *PAD3* activity contributes to the *dcl1*-conferred reduction in GPA fecundity.

As mentioned above, GPA as well as several other aphids also vector viral diseases. Genomes of many viruses encode suppressors of RNA silencing that impact plant defense signaling. For example, the 2b protein of cucumber mosaic virus (CMV) inhibits anti-viral miRNA silencing mechanisms, as well as interferes with JA and SA signaling (Ji and Ding, 2001; Lewsey et al., 2010). Since CMV is transmitted by GPA, CMV infection could potentially influence GPA performance on plants. Indeed, GPA survival was higher on CMV-infected tobacco, as compared to mock-infected plants (Ziebell et al., 2011). By comparison, to the mock- or CMV-infected tobacco, GPA survival was lower on tobacco plants infected with a *2b* gene-deletion mutant CMVΔ2b virus (Ziebell et al., 2011). EPG monitoring of GPA behavior confirmed that in comparison to insects feeding on plants infected with the WT CMV, GPA feeding on plants infected with the CMVΔ2b virus exhibited reduced feeding from sieve elements. Whether this effect of 2b on promoting GPA infection is due to its impact on RNA-silencing is not known. The impact of viral infection on aphid performance is not limited to GPA and CMV. By suppressing JA signaling in the host plant, the begomovirus *Tomato yellow leaf curl China virus* is beneficial to the insect vector, the whitefly (Zhang et al., 2012). Suppression of transcriptional silencing by the viral beta-satellite DNA-encoded factor βC1 was responsible for suppression of JA signaling during this tritrophic interaction. The interaction between Arabidopsis, the GPA and viruses vectored by GPA is also being utilized for high-throughput screens for Arabidopsis genes involved in this interaction (Chen et al., 2012).

### Roots as a source of a signal influencing arabidopsis-GPA interaction

In recent years, studies on the role of roots in plant responses to above-ground herbivory have gained momentum (Kaplan et al., 2008; Rasmann and Agrawal, 2008; Erb et al., 2009; Erb, 2012; Nalam et al., 2013b). For example, the secondary metabolite and insecticide nicotine is synthesized in the roots from where it is transported to the shoots to be stored in vacuoles and provide defense against herbivores (Morita et al., 2009). The maize Mir1-CP cysteine protease, which accumulates at elevated levels in leaves in response to fall armyworm (*Spodoptera frugiperda*) infestation, is also suggested to be synthesized in the roots from where it is transported to the shoots (Lopez et al., 2007). Very recently, Nalam et al. (2012) demonstrated that communication between the roots and shoots also has a critical role in the interaction between Arabidopsis and GPA. GPA feeding on Arabidopsis leaves resulted in the up-regulation of *LOX5* (*LIPOXYGENASE 5*) expression in roots. *LOX5* encodes a 9-LOX, which is involved in the oxidation of fatty acids to yield oxylipins that can be further modified to yield a variety of biologically active lipids. Levels of 9-LOX-derived oxylipins also increased in roots, shoots and petiole exudates of plants that had GPA on their shoots. Loss of *LOX5* function resulted in reduced colonization of the *lox5* mutant by GPA. Insects reared on the *lox5* mutant had significantly lower water content compared to insects reared on the WT plant. Irrigation with 9-LOX products restored water content and restored insect colonization on the *lox5* mutant, suggesting that a 9-LOX lipid or product thereof is required for facilitating GPA colonization on WT plants. EPG monitoring of insect feeding behavior indicated that on the *lox5* mutant, GPA spent less time feeding from sieve elements and drinking from the xylem, thus accounting for the reduced water content in the insects. Grafting experiments in which the WT shoots were grafted on *lox5* roots and *vice-versa* indicated that *LOX5* function is required in roots to promote GPA colonization on Arabidopsis shoots (Nalam et al., 2012).

A variety of LOX-derived oxylipins are known to function as defense modulating signaling molecules in plants. Similarly, a 9-LOX product could potentially be involved in suppressing Arabidopsis defense against GPA. Alternatively, GPA might have evolved to target the *LOX5* pathway to produce oxylipins that suppress and/or counter Arabidopsis defenses against GPA. When added to synthetic diet, 9-LOX products enhanced insect fecundity, suggesting that these lipids might be directly acting on the insect to stimulate feeding and simultaneously promote fecundity. Previously it was shown that GPA reared on plants contained 9-LOX-derived lipids, while insects reared on synthetic diet lacked these lipids (Gosset et al., 2009), suggesting that GPA derives these lipids from the host. Indeed, in comparison to GPA reared on WT Arabidopsis, which contained 9-LOX synthesized oxylipins, GPA reared on the *lox5* mutant lacked these lipids (Nalam et al., 2013a), supporting the notion that the GPA derives these 9-LOX synthesized lipids from Arabidopsis. The presence of 9-LOX products in the phloem sap-enriched petiole exudates of Arabidopsis supports this suggestion (Nalam et al., 2012). However, one cannot rule out the possibility that GPA synthesizes these oxylipins only in response to a plant-derived stimuli that is missing in the *lox5* mutant.

If 9-LOX lipids are susceptibility factors, then why should plants increase synthesis of 9-LOX products when colonized by GPA? Recently, Nalam et al. (2013a) suggested that root-synthesized 9-LOX lipids are engaged by the plant to activate defenses in the shoots. The GPA infestation-induced up-regulation of *PAD4* expression was attenuated in the *lox5* mutant (Nalam et al., 2013a). Furthermore, 9-LOX products when applied to WT Arabidopsis induced *PAD4* expression (Nalam et al., 2013a). Thus, as indicated in Figure 2, while Arabidopsis utilizes LOX5-derived lipids to activate defenses, the GPA has likely evolved to utilize 9-LOX-derived oxylipins, or products thereof, consumed from the host plant as a cue to facilitate feeding and promoting fecundity.

## Concluding remarks

Although for quite some time Arabidopsis has been utilized as a model plant to understand the molecular basis of plant growth, development and response to pathogens, it is only in the last 10 years that significant progress has been made on utilizing Arabidopsis to study plant interaction with phloem-sap consuming insects. The compatible interaction between Arabidopsis and GPA has provided new insights on the physiological and molecular adaptations that contribute to controlling the severity of infestation. In addition, this interaction has also begun to provide information on how the insect targets host mechanisms to facilitate colonization. The near completion of the GPA genome sequence (AphidBase; www.aphidbase.org) and the recent development of plant delivered RNAi approaches to silence GPA gene expression should now facilitate understanding the function of GPA genes in this interaction, as well.

### Conflict of interest statement

The authors declare that the research was conducted in the absence of any commercial or financial relationships that could be construed as a potential conflict of interest.
